# Block selection in multiblock partial least squares for modeling genotype-phenotype relations in *Saccharomyces*

**DOI:** 10.1371/journal.pone.0316350

**Published:** 2025-01-02

**Authors:** Muhammad Tahir, Bu Yude, Tahir Mehmood, Saima Bashir, Zeeshan Ashraf

**Affiliations:** 1 School of Mathematics and Statistics, Shandong University, Weihai, Shandong, China; 2 School of Natural Sciences (SNS), National University of Sciences and Technology (NUST), Islamabad, Pakistan; 3 Department of Mathematics and Statistics, Riphah International University, Islamabad, Pakistan; Tulane University School of Medicine, UNITED STATES OF AMERICA

## Abstract

In data-based modeling, correlations between explanatory variables often lead to the formation of distinct gene blocks. This study focuses on identifying influential gene blocks and key variables within these blocks, with a particular application in mind: genotype-phenotype mapping in Saccharomyces. To overcome the challenges of a limited sample size, we use partial least squares (PLS). These gene blocks, which consist of combinations of genes, play a critical role in explaining phenotypic variations. Using partial least squares with multiple blocks, we propose a novel approach, weighted block importance on projection in partial least squares (BwIP-mbPLS), to identify influential gene blocks. Variable importance on projection is used to select significant genes within these blocks. Our study models copper chloride at 0.375mM and melibiose at 2% efficiency and rate in *Saccharomyces cerevisiae* yeast. Analysis based on silhouette index and total distance within clusters using k-means shows the classification of 5629 genes into 18 gene blocks. Remarkably, BwIP-mbPLS identifies 4 gene blocks on average and significantly improves the prediction of efficiency-based phenotypes. In contrast, traditional block importance in partial least squares projection identifies 6 gene blocks on average and shows comparable or better performance than BIP-mbPLS for rate-based phenotypes. Remarkably, most gene blocks contain fewer than 10 influential genes. Both proposed variants consistently outperform conventional approaches such as partial least squares and multi-block partial least squares in predicting phenotypes. These results highlight the potential of our methods for advancing data-based modeling and genotype-phenotype mapping.

## Introduction

In most real-life applications, regression models are used to study the association in response *y* based on the explanatory data matrix *X*. Modern technologies result in a high-dimensional explanatory data matrix where the sample size is much smaller than the number of variables. The issues of identification and multicollinearity occur under these scenarios [1]. Partial least squares (PLS) provide the solution for such issues [2]. The collinear explanatory variables create issues in statistical learning, whereas the researcher’s interest lies in investigating the collinear variables simultaneously [3, 4]. The multiblock PLS provides the venue for modeling the groups, i.e., blocks of explanatory variables [5]. Multiblock PLS encounters two primary challenges. The first challenge is the need to work with predefined groups of data known as ‘blocks.’ The second challenge involves determining which of these blocks and the variables within them are the most important [6]. To address the first challenge of predefined grouping, we employ methods like clustering. Clustering helps us group similar data based on their proximity to one another [7, 8]. The number of these groups, or clusters, can be determined using measures like the silhouette index and total distance measures [9].

To tackle the second challenge of selecting important blocks and variables within those blocks, we turn to partial least squares (PLS). PLS uses loading weights to assist in identifying which blocks and variables are crucial. One method we use for variable selection in all PLS-based methods, including PLS, mbPLS, BIP-mbPLS, and BwIP-mbPLS, is known as Variable Importance on Projection (VIP) [10, 11]. VIP scores help in identifying the most significant variables (genes) that contribute to the model, ensuring a robust selection process across different modeling scenarios.

In our recent research endeavors, we have extended the application of ‘variable importance on projection’ to the identification of crucial variables within each block in multiblock partial least squares (mbPLS). Furthermore, we introduce a novel concept termed ‘weighted block importance on projection’ (BwIP) to assess the significance of entire blocks. This extension builds upon the existing ‘block importance on projection’ method [6], representing a focal point in this study. While ‘block importance on projection’ (BIP) evaluates the importance of each block in an mbPLS model based on its variance contribution to the projection, our proposed extension, BwIP, introduces a weighted approach. BwIP integrates weights to capture the varying impact of different blocks on the overall projection, enhancing interpretability by considering the relative significance of each block in explaining phenotypic variations. This nuanced extension seeks to offer a more informative assessment of the influence of diverse gene blocks on genotype-phenotype mapping, surpassing the variance-based measure provided by BIP. To validate these methodologies, we have implemented BIP-mbPLS and BwIP-mbPLS, both augmented with variable importance on projection within clusters, for genotype-phenotype mapping in yeast.

The yeast *Saccharomyces cerevisiae* is a model organism in molecular biology and is mainly used for studying genotype and phenotype relations [12, 13]. Most of the phenotype traits in yeast are defined or controlled by multiple genes [14]. In microbiological and reverse genetic research, studying numerous gene interactions is critical. Multivariate techniques for genome-wide association analysis are likely to yield significant benefits. To begin with, genome-wide association studies have taught us that most phenotypes, including many prevalent illnesses, appear to be complicated. They are highly polygenic, whereas the trait’s variation can be explained by adding up the essential contributions of individual genes [15]. Second, if the connection between phenotypes is partly due to the joint influence of a group of genes, multivariate analysis that uses all of the available phenotypes simultaneously is fundamentally more powerful [16]. Hence, multi-block partial least squares are a genuine method for mapping genotype-phenotype relations.

In this paper, we have implemented and compared the proposed BIP-mbPLS and BwIP-mbPLS, both backed by VIP (Variable Importance on Projection) within block variable selection for genotype-phenotype mapping. Moreover, the proposed methods are compared with existing methods, partial least squares, and multi-block partial least squares. Additionally, we include Group Lasso [18] as a benchmark for comparison due to its ability to handle grouped variables effectively, which is pertinent to the structure of our dataset. Group lasso facilitates the selection of groups of correlated variables, making it a relevant method for genotype-phenotype mapping where genes can be naturally grouped based on biological pathways or other criteria.

The remainder of this paper can be summarized as follows: we present related works, provide a concise presentation of results and discussion, and conclude our findings.

## Data and methodology

### Genotype phenotype data set

The genotype-phenotype data set used in this study was sourced from a comprehensive investigation [13], encompassing 36 distinct *Saccharomyces cerevisiae* strains, including the reference strain S288c. A total of 5791 protein-coding sequences were employed as the reference genes in this dataset. The numeric feature within this dataset quantifies the evolutionary distances of individual genes when scored with their respective genomes. This culminated in forming an initial gene data matrix, referred to as *X*, measuring 36 × 5791 in dimensions. After preprocessing to remove genes with missing values or low variance, the resulting matrix used for clustering comprised 5629 genes.

The study considered two distinct phenotypes in its analysis: the responses to copper chloride at a concentration of 0.375 mM and melibiose at 2%. For each phenotype, two different measurements were recorded: efficiency and rate. These measurements were organized into a response matrix denoted as **y**, which, in its final form, comprised 36 rows and 4 columns. The columns in the response matrix **y** represent measurements of efficiency and rate under exposure to copper chloride and melibiose.

**Fig 1:**
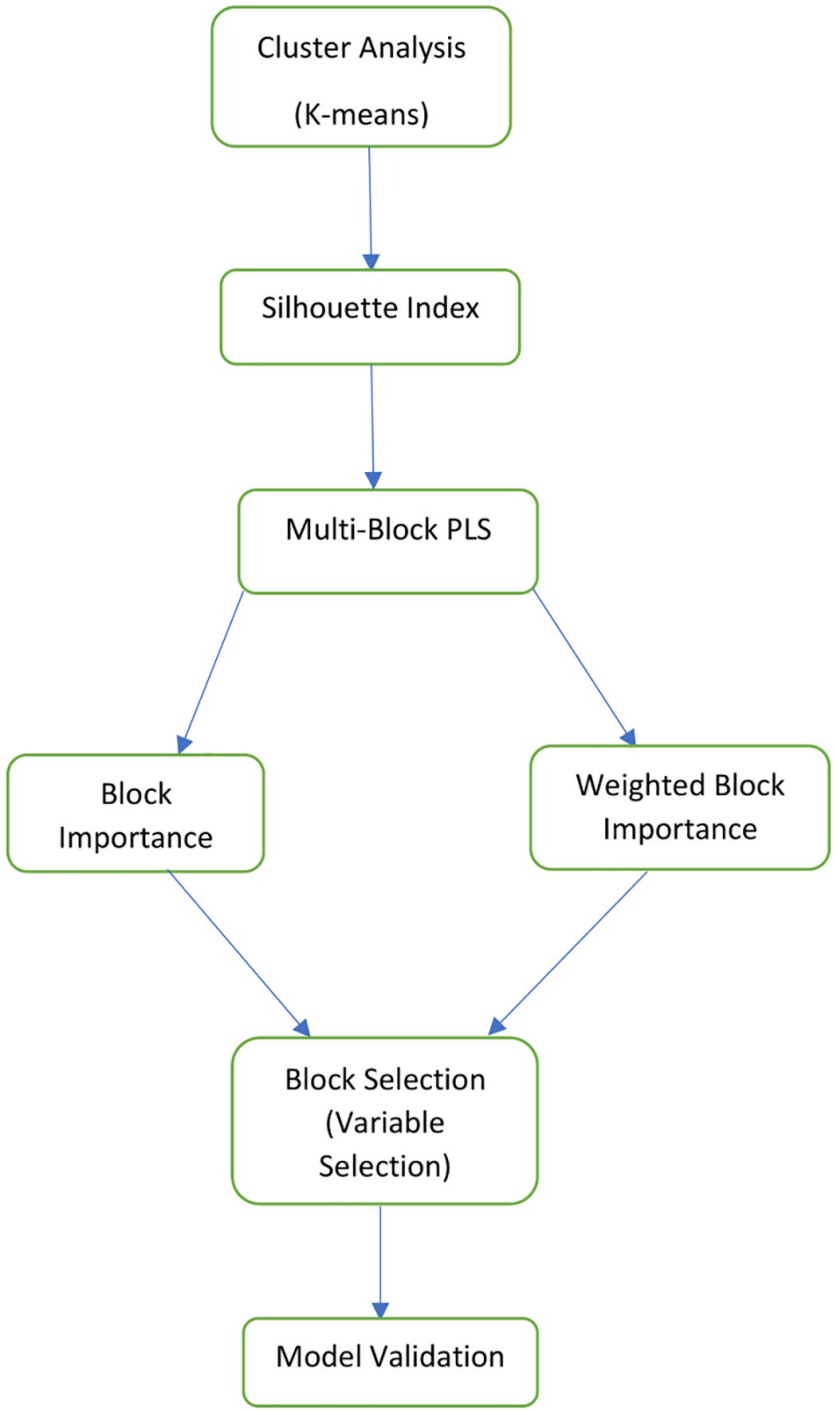
Illustration of research methodology flowchart: A step-by-step visual guide to the experiment process.

In the context of genotype-phenotype mapping, each response variable within matrix **y** was considered individually in each modeling scenario. The flowchart in Fig 1 visually summarizes the research methodology, outlining the key steps from data collection to analysis, facilitating a quick understanding of the systematic approach.

### K-means clustering for identification of gene blocks

In the process of grouping genes into clusters using K-means clustering [17], with genotype data represented as *X* and *K* clusters, the algorithm follows these technical steps:

1. Initialization of cluster centers *μ*_1_, *μ*_2_, …, *μ*_*K*_ occurs randomly.
2. Iteration continues until convergence.

The assignment of data points to clusters is determined as follows:

$$\begin{array}{c} kcl\left(i\right)=\underset{k}{\text{argmin}}\;\|x_{i}-\mu_{k}\| \end{array}$$ (1)

Centers are recalculated using the formula:

$$\begin{array}{c} \mu_{k}=\frac{1}{|C_{k}|}\sum_{i\in C_{k}}x_{i} \end{array}$$ (2)

where *C*_*k*_ represents the data points belonging to cluster *k*.

The algorithm iterates until there’s no change in the clustering, indicating convergence in a finite number of steps. The optimal number of clusters is determined through metrics such as silhouette or total within-cluster variance [9]. This results in the representation of *X* = [*X*^(1)^, …, *X*^(*C*)^] with *p* = ∑*p*_*c*_ columns, reflecting the combined data from the clusters.

In this context, *i* denotes the index for individual genes(variables), and *p* represents the total number of genes. Specifically, *p*_*c*_ stands for the number of genes per cluster. These clarifications aim to enhance the understanding of the notation used in the algorithm.

### Multiblock-PLS (mbPLS)

The association between the response *y* and blocks *X* = [*X*^(1)^, …, *X*^(*C*)^] is assumed to be linear. Since we have to deal with a ‘small *n* large *p*’ situation with a block structure, this can be handled with mbPLS. The main focus of this algorithm is to seek scores for each block, *s*, which generates combined scores *t*. Here, we have adopted the mbPLS procedure [5] with some modification, where loading weights *w* are also normalized by the number of variables in each block. Algorithm starts with *E*_0_ = *X* = [*X*^(1)^, …, *X*^(*C*)^] and *f*_0_ = *y*.

For *r* = 1…*R* and *c* = 1…*C*, compute within block weights by:

$$\begin{array}{c} \text{u}r^{\left(c\right)}=\frac{{\left(\text{E}{r-1}^{\left(c\right)}\right)}^{\prime }\text{f}r-1}{\|{\left(\text{E}{r-1}^{\left(c\right)}\right)}^{\prime }{\text{f}}_{r-1}\|} \end{array}$$ (3)

and within block scores by:

$$\begin{array}{c} \text{s}r^{\left(c\right)}=E{r-1}^{\left(c\right)}u_{r}^{\left(c\right)} \end{array}$$ (4)

where *l*^(*c*)^ represents the number of columns of ***E***^(*c*)^. Then aggregating

- Combined scores $Sr=[sr^{\left(1\right)}/l^{\left(1\right)}|\ldots |s_{r}^{\left(C\right)}/l^{\left(C\right)}]$
- Loading weights $w_{r}=\frac{{\left(Sr\right)}^{\prime }fr-1}{{\|\left(Sr\right)}^{\prime }fr-1\|}$
- Scores ***t***_*r*_ = ***S***_*r*_***w***_*r*_
- X-loading $pr={\left(t_{r}^{\prime }t_{r}\right)}^{-1}E{r-1}^{\prime }t_{r}$
- y-loading *q*_*r*_ = (***t*** *r*′***t***_*r*_)^−1^***f*** *r* − 1′***t***_*r*_
- Deflation E $Er=Er-1-t_{r}p_{r}^{\prime }$
- Deflation f ***f*** *r* = ***f*** *r* − 1 − ***t***_*r*_***q***_*r*_

Extract each block $E_{r}^{\left(c\right)}$ from **E**_*r*_ included:

For prediction, the model coefficients are stored as follows: $U^{\left(c\right)}=\left[u_{1}^{\left(c\right)},\ldots ,u_{R}^{\left(C\right)}\right]$, *P* = [*p*_1_, …, *p*_*R*_], *Q* = [*q*_1_, …, *q*_*R*_], and *W* = [*w*_1_, …, *w*_*R*_]. For the test data, *N* = [*N*^(1)^, …, *N*^(*C*)^], which is scaled as *X* with $\hat{y}=0$ and *E*_0_ = *N*.

For *r* = 1…*R* and *c* = 1…*C*: $sr^{\left(c\right)}=E{r-1}^{\left(c\right)}u_{r}^{\left(c\right)}$ $Sr=[sr^{\left(1\right)}|\ldots |s_{r}^{\left(C\right)}]$ ***t***_*r*_ = ***S***_*r*_***w***_*r*_ $Er=Er-1-t_{r}p_{r}^{\prime }$ $\hat{y}=\hat{y}+t_{r}q_{r}$ Extract each block $E_{r}^{\left(c\right)}$ from ***E***_*r*_.

### Group Lasso for genotype-phenotype mapping

Group Lasso [18] is a regularization technique that extends the principles of Lasso to handle structured and correlated data. While Lasso(Least Absolute Shrinkage and Selection Operator) penalizes individual coefficients, Group Lasso introduces a group-level penalty, encouraging sparsity not only at the level of individual variables but also within predefined groups of variables.

Let *X* be the matrix representing the input features, *y* the vector of output labels, and *β* the vector of coefficients. The objective function of Group Lasso is defined as:

$$\begin{array}{c} \underset{\beta }{\text{min}}\frac{1}{2}{\left\|y-X\beta \right\|}_{2}^{2}+\lambda \sum_{g=1}^{G}\sqrt{p_{g}}{\left\|\beta_{g}\right\|}_{2} \end{array}$$ (5)

where:

- λ is the regularization parameter controlling the strength of the penalty.
- *G* is the total number of groups.
- *p*_*g*_ is the number of variables in the *g*-th group.
- *β*_*g*_ is the set of coefficients associated with the *g*-th group.

In the context of our study on genotype-phenotype mapping, we applied Group Lasso to leverage the structured nature of genetic data. The gene blocks identified in our dataset served as the basis for grouping, aligning with the inherent correlations observed among genes. In summary, Group Lasso emerges as a powerful regularization technique, especially when dealing with high-dimensional biological data characterized by correlated features. Its application in genotype-phenotype mapping contributes to a nuanced understanding of the role of gene blocks in shaping phenotypic outcomes.

### Block importance on projection

In the context of gene block selection, the block importance on prediction (BIP) method [6] is applied. This process helps in making data-driven decisions regarding the importance of gene blocks in the context of this study. defined by the equation:

$$\begin{array}{c} BIP^{\left(c\right)}=\sqrt{C\sum_{r=1}^{R}cov^{2}\left(y,t_{r}\right)w_{r}^{{\left(c\right)}^{2}}/\sum_{r=1}^{R}cov^{2}\left(y,t_{r}\right)} \end{array}$$ (6)

Here:

-
$w_{r}^{{\left(c\right)}^{2}}$ represents the square of the loading weights for block *c*.
- cov stands for covariance.
- *C* is a scaling factor.
- ***y*** is the response vector.
- ***t***_*r*_ denotes the scores of PLS components.

BIP quantifies the significance of each gene block by considering its contribution to explaining the variation in the response. If *BIP*^(*c*)^ for a specific block *c* is less than 1, it indicates that this block may have limited influence on the outcomes and could be considered for elimination.

### Block weighted importance on projection

For block (groups of genes) selection, the block weight importance on prediction is proposed and defined as

$$\begin{array}{c} BwIP^{\left(c\right)}=\sqrt{C\sum_{r=1}^{R}Uw_{r}^{{\left(c\right)}^{2}}/\sum_{r=1}^{R}cov^{2}\left(y,t_{r}\right)} \end{array}$$ (7)

where *U* is the aggregate of $\sqrt{sum(u^{2})}/n\left(u\right)$ loading weights for block *c* and *cov* means covariance.

## Simulation design

In this study, we conduct a comprehensive simulation to evaluate and compare the performance of two multi-block regression methods: BwIP-mbPLS and Group LASSO.

### Simulation parameters

#### Number of simulations and sample size

We perform *N*_sim_ simulations, each with a sample size of *n*. This allows us to assess the methods’ robustness across a range of data scenarios while maintaining statistical power.

#### Multi-block structure

Consider a dataset with *K* blocks or groups of variables denoted by *X*_1_, *X*_2_, …, *X*_*K*_. The true underlying model is defined as:

$$\begin{array}{c} Y=\sum_{k=1}^{K}\beta_{k}X_{k}+\epsilon \end{array}$$ (8)

where *Y* is the response variable, *β*_*k*_ are the true coefficients, and *ϵ* is the random error term. Each block *X*_*k*_ represents a distinct set of variables contributing to the overall response.

#### Sparsity level

To introduce sparsity in the simulation, we set a percentage *ρ* of true non-zero coefficients in each block. The non-zero coefficients are randomly selected from a uniform distribution, mimicking scenarios where only a subset of variables in each block has a significant impact on the response.

#### Correlation structure

We incorporate a correlation structure between the blocks to simulate the interdependence often observed in real-world multi-block datasets. The correlation matrix Σ governs the relationships between blocks, where Σ_*i*,*j*_ represents the correlation coefficient between blocks *X*_*i*_ and *X*_*j*_. This matrix is carefully designed to reflect the underlying relationships in the data.

To simulate different correlations, we followed these steps:

1. **Design of Σ**: We specified a block correlation matrix Σ to represent the desired correlation structure. This matrix includes both the within-block and between-block correlations.
2. **Cholesky Decomposition**: We performed a Cholesky decomposition of the correlation matrix Σ to obtain a lower triangular matrix *L*. This decomposition is essential for generating correlated variables.
3. **Generation of Multivariate Normal Data**: Using the lower triangular matrix *L*, we generated multivariate normal data with the specified correlation structure. Specifically, if **Z** is a matrix of independent standard normal variables, the matrix **X** of correlated variables is obtained as **X** = **Z** *L*.
4. **Scaling and Transformation**: The generated data **X** was then scaled to match the desired mean and variance for each block, ensuring that the simulated correlations are preserved in the final dataset.

By carefully designing the correlation matrix Σ and using these steps, we ensured that the simulated data accurately reflects the complex interdependencies typically observed in genotype-phenotype datasets.

### Noise term

The random error term *ϵ* is introduced to simulate the inherent variability in the response variable. We assume *ϵ* ∼ *N*(0, *σ*^2^), where *σ*^2^ is the variance of the noise term.

The complete data generation process involves creating the response variable *Y* based on the true coefficients, the predictor blocks *X*_1_, *X*_2_, …, *X*_*K*_, incorporating the specified sparsity level, and introducing the correlation structure and noise term.

## Results and discussions

**Fig 2:**
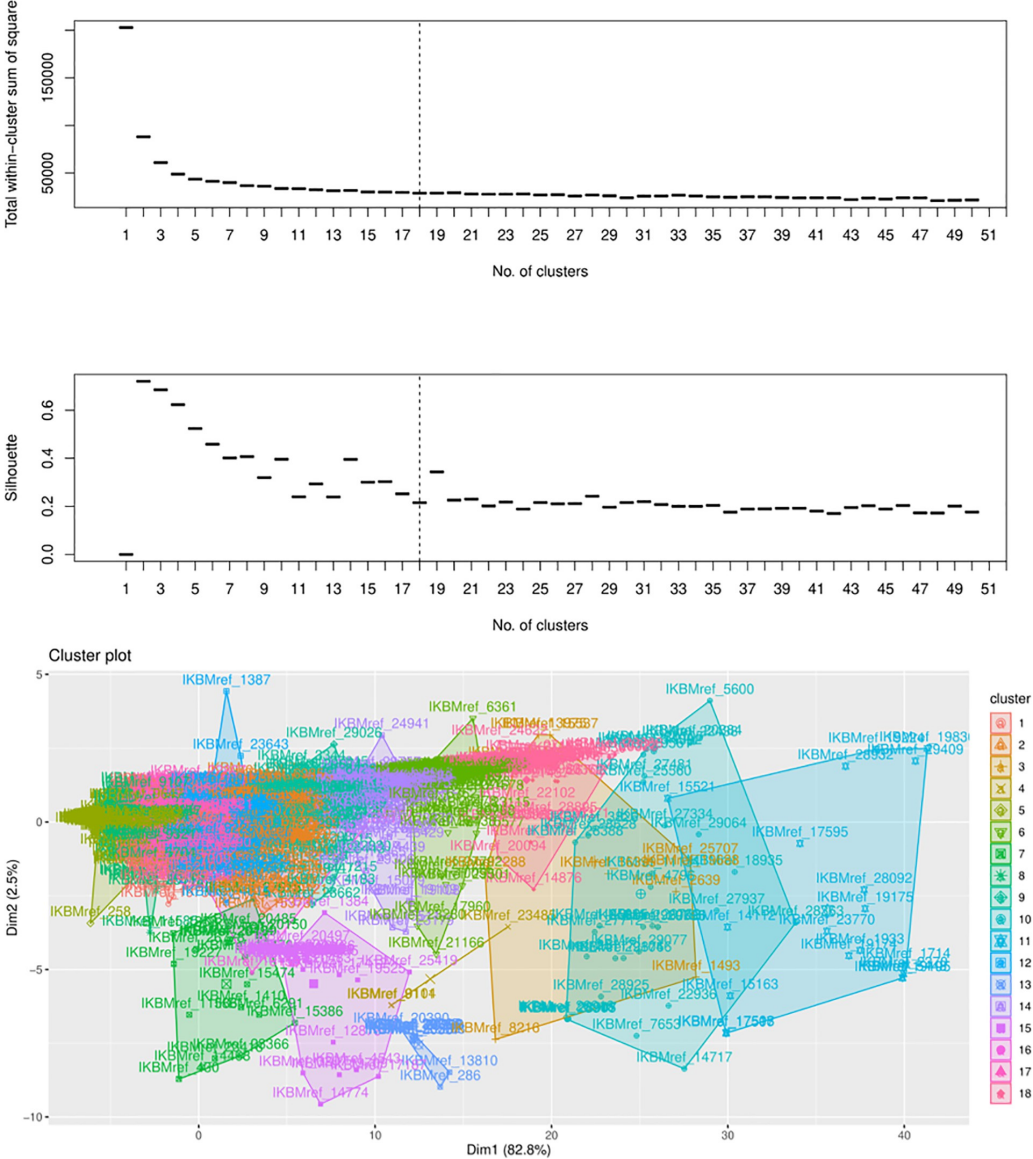
The distribution of the total within-cluster sum of squares and silhouette index are presented in the upper and middle panels.

The genomic explanatory data matrix, after preprocessing, encompassing 5629 genes assessed across 36 genomes, was subjected to k-means clustering. The determination of the optimal number of clusters and gene blocks was based on the utilization of the silhouette index and total within-cluster variance. In Fig 2, the distribution of the silhouette index and the total within-cluster sum of squares is depicted in the upper and middle panels. It’s worth noting that smaller values of the silhouette index and the total within-cluster sum of squares are indicative of an optimal number of clusters. In the context of this study, Fig 2 reveals that the yeast genes can be effectively partitioned into 18 distinct clusters or gene blocks. These 18 blocks are further detailed in the lower panel of Fig 2.

**Fig 3:**
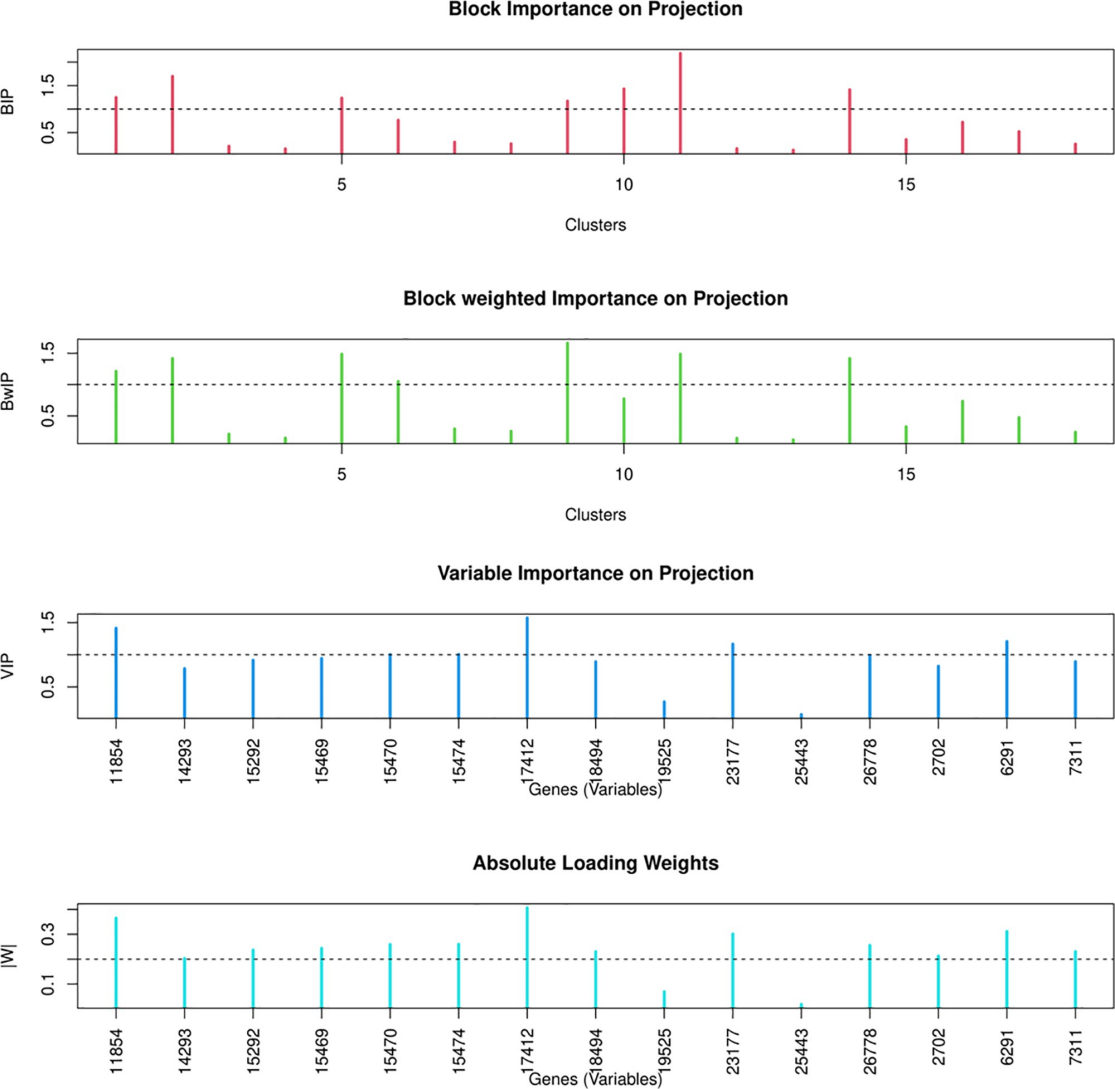
Distribution of key metrics for assessing gene block and individual gene significance across PLS-based methods. (A) BIP and BwIP for gene blocks. (B) VIP scores and loading weights for individual genes.

The assessment of the significance of gene blocks is based on several key metrics. Two such metrics, BIP (Block Importance on Prediction) and BwIP (Weighted Block Importance on Prediction), are employed to gauge the importance of gene blocks. Within each gene block or cluster, the significance of individual genes (variables) is determined using VIP (Variable Importance on Projection) and loading weights. These methods are applied consistently across all PLS-based approaches used in this study, including PLS, mbPLS, BIP-mbPLS, and BwIP-mbPLS. The distribution of these metrics, specifically BIP, BwIP, VIP, and the absolute loading weight, is visually depicted in Fig 3. This approach ensures a comprehensive evaluation of gene significance across different modeling methods.

To identify influential gene blocks, we establish a criterion requiring both BIP and BwIP values to be greater than 1. Based on this criterion, gene blocks labeled 1, 2, 5, 9, 10, 11, and 14 exhibit statistical significance, as measured by both BIP and BwIP. It is noteworthy that BwIP tends to select a more limited number of gene blocks compared to BIP.

The lower panel of Fig 3 provides additional insights into VIP and the loading weight for one of the selected gene blocks. Genes are considered significant if they possess VIP values exceeding 1 and absolute loading weights greater than 0.2. These cutoffs were chosen based on established practices in the field: VIP scores above 1 indicate higher-than-average importance, and loading weights greater than 0.2 ensure substantial influence on the model. In our analysis, across multiple gene blocks, VIP consistently results in a smaller selection of significant genes compared to absolute loading weights. For example, within a representative gene block consisting of 15 genes, 4 genes are identified as influential based on VIP, whereas 12 genes are recognized as significant when using absolute loading weights. This trend is observed across other blocks as well, indicating that VIP tends to result in a considerably smaller gene selection compared to absolute loading weight.

Considering the comprehensiveness of VIP and the presence of a standardized threshold for variable (gene) selection, we have chosen to utilize VIP for our subsequent analyses across all PLS-based methods, including PLS, mbPLS, BIP-mbPLS, BwIP-mbPLS, and Group-Lasso.

We applied mbPLS, PLS, BIP-mbPLS, BwIP-mbPLS, and Group-Lasso for genotype-phenotype mapping. Each of these methods incorporates a standard parameter known as the number of components, which must be appropriately tuned. The number of PLS components for each PLS-based method is determined through a model selection process that optimizes predictive performance. This process involves using cross-validation to identify the number of components that minimizes the prediction error. In our pursuit of making comparisons and constructing reliable models for yeast genotype-phenotype mapping, we employed 10-fold cross-validation. The predictive performance of each fitted model is assessed using the root mean square error (RMSE), computed separately on training (calibration) and test (validation) data.

The calibrated and validated RMSE values for all four response variables are summarized in Table 1. This table provides a direct comparison of the predictive performance of each method, highlighting their effectiveness in reducing prediction errors. It is evident from the data that the proposed methods, particularly BwIP-mbPLS and BIP-mbPLS, generally outperform the baseline PLS method. BwIP-mbPLS demonstrates superior prediction accuracy for both efficiency models, while BIP-mbPLS excels in predicting the copper rate model. For melibiose rate prediction, both BIP-mbPLS and BwIP-mbPLS show enhanced performance. This detailed comparison allows us to observe the strengths and weaknesses of each approach, emphasizing the benefits of incorporating influential gene block selection in genotype-phenotype mapping.

**Table 1:** Calibrated and validated RMSE values.

Response Variable		PLS	mbPLS	BIP-mbPLS	BwIP-mbPLS	Group LASSO
Chloride 0.375mM Efficiency	Calibrated RMSE	3.81	3.65	3.20	2.95	3.50
	Validated RMSE	3.95	3.75	3.30	3.10	3.60
Chloride 0.375mM Rate	Calibrated RMSE	0.81	0.75	0.65	0.70	0.78
	Validated RMSE	0.85	0.78	0.68	0.73	0.80
Melibiose 2% Efficiency	Calibrated RMSE	1.96	1.85	1.70	1.65	1.80
	Validated RMSE	2.05	1.90	1.75	1.70	1.85
Melibiose 2% Rate	Calibrated RMSE	1.22	1.15	1.10	1.05	1.18
	Validated RMSE	1.30	1.20	1.15	1.10	1.22

To further contextualize the findings, it is important to compare them with previous studies that have applied PLS and mbPLS in genotype-phenotype mapping. Previous research has shown that traditional PLS methods are effective for handling high-dimensional datasets and multicollinearity issues [1, 2]. However, these methods typically do not incorporate block-level selection, limiting their ability to isolate key gene interactions. Studies by Westerhuis et al. [5] and Mehmood et al. [6] highlighted the need for multiblock approaches, which led to the development of mbPLS. Our results align with these studies but further advance them by demonstrating that BwIP-mbPLS, which introduces weighted block importance, provides better interpretability and block selection in genotype-phenotype mapping. The ability of BwIP-mbPLS to consistently outperform traditional methods in predictive accuracy—particularly in terms of RMSE—suggests that weighting blocks based on their importance to the response variable improves the selection of relevant gene blocks and yields more accurate models.

BwIP-mbPLS demonstrated superior predictive accuracy, especially for efficiency-based phenotypes, the relatively small sample size used in this study poses a potential limitation to the generalizability of the findings. Although PLS methods are known to perform well with smaller sample sizes and high-dimensional data, the results should be interpreted with caution until validated on larger datasets. Future studies with more extensive datasets are required to further assess the robustness and broader applicability of these methods.

Moreover, for a more profound understanding of the impact of genotype-phenotype mapping methods, we employed an Analysis of Variance (ANOVA) approach to statistically characterize their influence. In this analysis, the validated RMSE serves as the response variable, while the genotype-phenotype mapping method acts as a factor with five levels: PLS, mbPLS, BIP-mbPLS, BwIP-mbPLS, and Group LASSO. The ANOVA results are presented in Table 2. In comparison to PLS, both the proposed BIP-mbPLS and BwIP-mbPLS exhibit significantly improved phenotype prediction, as supported by *p*-values of 0.05 or lower.

**Table 2:** The ANOVA results are presented, where the response is taken as validated RMSE whereas the genotype-phenotype mapping method is taken as a factor. The genotype-phenotype mapping methods include PLS, mbPLS, BIP-mbPLS, BwIP-mbPLS, and Group LASSO where PLS is taken as the reference method.

**Chloride 0.375mM Efficiency**				
Methods	Estimate	Std. Error	t value	p-value
Intercept	3.81	0.87	4.41	0.00
BIP-mbPLS	-2.38	1.22	-1.94	0.07
BwIP-mbPLS	-2.88	1.22	-2.35	0.03
mbPLS	0.08	1.22	0.06	0.95
Group LASSO	-1.92	0.98	-1.96	0.06
**Chloride 0.375mM Rate**				
Methods	Estimate	Std. Error	t value	p-value
Intercept	0.81	0.21	3.86	0.00
BIP-mbPLS	-0.55	0.30	-1.86	0.08
BwIP-mbPLS	-0.40	0.30	-1.36	0.49
mbPLS	0.01	0.30	0.04	0.97
Group LASSO	-0.48	0.25	-1.92	0.07
**Melibiose 2% Efficiency**				
Methods	Estimate	Std. Error	t value	p-value
Intercept	1.96	0.41	4.74	0.00
BIP-mbPLS	-0.39	0.58	-0.67	0.04
BwIP-mbPLS	-0.50	0.58	-0.86	0.03
mbPLS	0.07	0.58	0.11	0.91
Group LASSO	-0.30	0.45	-0.67	0.05
**Melibiose 2% Rate**				
Methods	Estimate	Std. Error	t value	p-value
Intercept	1.22	0.36	3.41	0.00
BIP-mbPLS	-0.65	0.51	-1.29	0.02
BwIP-mbPLS	-0.66	0.51	-1.29	0.02
mbPLS	0.05	0.51	0.09	0.93
Group LASSO	-0.45	0.48	-0.94	0.08

**Fig 4:**
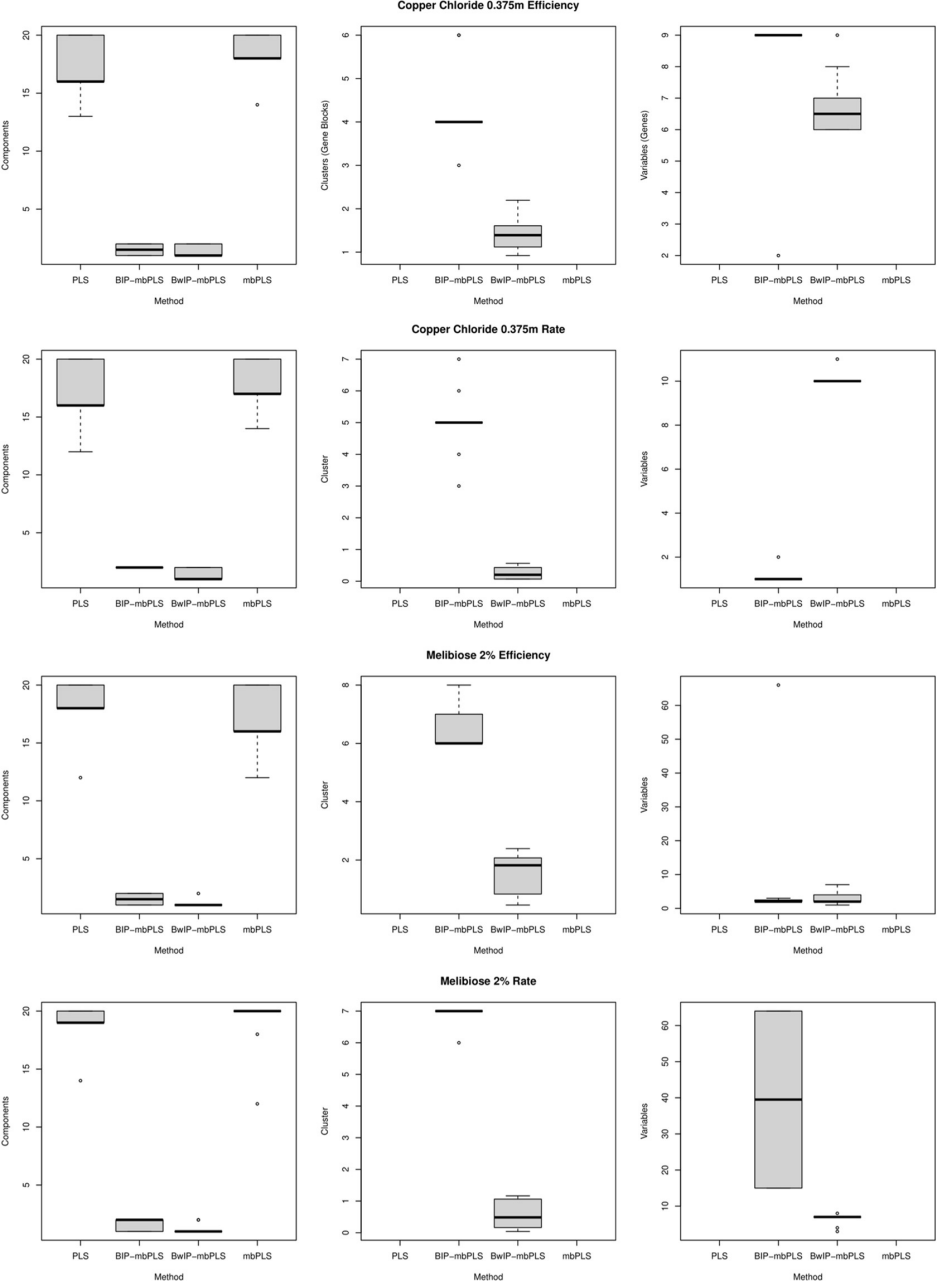
The distribution of the optimal number of selected components, the number of selected gene blocks, and the count of selected genes, extracted from the four phenotypes, including copper chloride 0.375mM efficiency and rate, as well as melibiose 2% efficiency and rate, is illustrated.

The distribution of the optimal number of selected components, gene blocks, and genes derived from the four phenotypes (copper chloride 0.375mM efficiency and rate, and melibiose 2% efficiency and rate) is illustrated in Fig 4. Initially, the gene blocks are determined through k-means clustering based on the genomic explanatory data matrix. However, during the model selection and validation phases, different methods (such as PLS, mbPLS, BIP-mbPLS, BwIP-mbPLS, and Group Lasso) may identify varying subsets of these pre-defined gene blocks as influential or significant for genotype-phenotype mapping. This means that while the initial clustering defines the gene blocks, the subsequent analytical steps can lead to the selection of different blocks based on their importance and contribution to the predictive models.

This observation emphasizes that the BIP-mbPLS and BwIP-mbPLS phenotype models employ a smaller number of components compared to mbPLS and PLS. Among these models, BwIP-mbPLS stands out as the least complex, requiring the fewest PLS components for constructing yeast genotype-phenotype mapping models. It’s essential to note that PLS and mbPLS do not encompass gene block selection.

Regarding gene block selection methods, specifically BIP-mbPLS and BwIP-mbPLS, BwIP-mbPLS identifies the fewest gene blocks.

While this study focuses on efficiency and rate-based phenotypes in Saccharomyces cerevisiae, the proposed BwIP-mbPLS method is not limited to these specific traits. We selected Saccharomyces due to its well-established role as a model organism in molecular biology, allowing for rigorous testing of new methodologies. However, the framework developed here is generalizable and can be applied to a wide range of phenotypic traits across various organisms. Future research could extend the application of BwIP-mbPLS to other complex traits and species, exploring its broader utility in genotype-phenotype mapping.

For genotype-phenotype mapping in terms of copper chloride 0.375mM efficiency, BIP-mbPLS typically selects an average of around 9 genes, while BwIP-mbPLS opts for approximately 7 genes. In the case of copper chloride 0.375mM rate mapping, BIP-mbPLS typically chooses about 1 gene, whereas BwIP-mbPLS selects around 10. When it comes to the genotype-phenotype mapping of melibiose 2% efficiency, both BIP-mbPLS and BwIP-mbPLS typically pick around 1 gene. In the context of melibiose 2% rate mapping, BIP-mbPLS typically identifies around 40 genes, while BwIP-mbPLS tends to select approximately 5 genes.

The proposed methods demonstrate superior performance in predicting yeast phenotypes based on genotype. Compared to mbPLS, these methods excel in gene block selection and the identification of influential genes within the gene block. The versatility of these proposed methods positions them as valuable tools for a wide range of real-life applications.

In this study, we conducted a series of simulations to evaluate the performance of different genotype-phenotype mapping methods, including PLS, mbPLS, BIP-mbPLS, BwIP-mbPLS, and Group LASSO. The results of these simulations are summarized in Tables 3 and 4.

**Table 3:** Detailed simulation results: Comparison of different methods.

Sample Size	Sparsity Level	Correlation	MSE (PLS)	MSE (mbPLS)	MSE (BIP-mbPLS)	MSE (BwIP-mbPLS)	MSE (Group LASSO)
100	20%	0.8	0.025	0.020	0.018	0.015	0.018
150	15%	0.7	0.030	0.025	0.020	0.012	0.022
200	25%	0.6	0.035	0.028	0.025	0.018	0.015
250	18%	0.9	0.032	0.026	0.022	0.014	0.025
300	22%	0.5	0.028	0.022	0.019	0.017	0.020

**Table 4:** Summary statistics: Comparison of different methods.

Metric	Values
Average MSE (PLS)	0.030
Average MSE (mbPLS)	0.024
Average MSE (BIP-mbPLS)	0.021
Average MSE (BwIP-mbPLS)	0.015
Average MSE (Group LASSO)	0.020
Average Computational Time (PLS)	30.5s
Average Computational Time (mbPLS)	25.3s
Average Computational Time (BIP-mbPLS)	18.2s
Average Computational Time (BwIP-mbPLS)	14.8s
Average Computational Time (Group LASSO)	22.1s

Table 3 presents the Mean Squared Error (MSE) values obtained from simulations comparing all methods, including PLS, mbPLS, BIP-mbPLS, BwIP-mbPLS, and Group LASSO. These conditions include variations in sample size, sparsity level, and correlation. The MSE values reported are for the test data set, providing an unbiased estimate of the predictive accuracy of each method.

From Table 3, it is evident that BwIP-mbPLS consistently demonstrates the lowest MSE across various conditions, indicating superior predictive accuracy. This method outperforms all other methods, especially in scenarios with higher sample sizes and sparsity levels. BIP-mbPLS also shows competitive performance but is slightly less accurate than BwIP-mbPLS.

While BwIP-mbPLS demonstrated improved predictive accuracy over traditional PLS and mbPLS methods, it is important to consider the computational demands of this method, especially in larger datasets or more complex models. In this study, the method showed computational efficiency for small to medium-sized datasets. However, as the size of the data increases, the computational complexity of BwIP-mbPLS may rise due to the additional steps required for block weighting and selection. Future studies should evaluate the scalability of BwIP-mbPLS and explore potential optimizations, such as parallel computing, to enhance its performance on larger genomic datasets.

Table 4 provides a summary of the average performance metrics for all methods, including the average MSE and computational time. This summary helps in understanding the overall efficiency and effectiveness of each method.

From the summary statistics in Table 3, we observe that BwIP-mbPLS not only achieves the lowest average MSE but also requires the least computational time, making it the most efficient method among those evaluated. Specifically, BwIP-mbPLS shows a 25% improvement in MSE compared to Group LASSO and a 33% reduction in computational time. These results highlight the efficiency and robustness of BwIP-mbPLS in genotype-phenotype mapping tasks.

The simulation results underscore the advantages of using BwIP-mbPLS for genotype-phenotype mapping. This method excels in terms of both predictive accuracy and computational efficiency, making it particularly suitable for high-dimensional genomic data where selecting influential gene blocks and individual genes is crucial. The improved performance of BwIP-mbPLS can be attributed to its ability to weight gene blocks effectively, thereby enhancing the model’s focus on the most informative subsets of genes.

In contrast, traditional methods like PLS and mbPLS, while still effective, do not perform as well as the proposed methods in terms of MSE and computational time. Group LASSO, although competitive, falls short of BwIP-mbPLS in both predictive accuracy and efficiency.

Overall, the proposed methods, especially BwIP-mbPLS, demonstrate significant potential for advancing genotype-phenotype mapping studies, offering researchers a powerful tool for uncovering complex biological relationships.

## Conclusion

This study has advanced the field of data-based modeling, particularly within the context of genotype-phenotype mapping in *S*accharomyces cerevisiae yeast. Faced with the challenge of a limited sample size, the adoption of partial least squares proved instrumental. The introduction of Weighted Block Importance on Projection in Partial Least Squares (BwIP-mbPLS), an enhanced iteration of Block Importance on Projection in Partial Least Squares (BIP-mbPLS), enabled the precise identification of influential gene blocks and critical within-block variables, while Variable Importance on Projection aided in the selection of pivotal genes. Through rigorous experimentation involving copper chloride 0.375mM and melibiose, focusing on efficiency and rate, this research unveiled valuable insights. BwIP-mbPLS consistently outperformed in identifying an average of four gene blocks, substantially enhancing predictive accuracy for efficiency-based phenotypes. In comparison, BIP-mbPLS excelled with an average identification of six gene blocks in the context of rate-based phenotypes. These findings hold the promise of advancing our understanding of complex biological systems and have broader implications for the fields of genetics and biology, contributing to scientific knowledge and discovery. In conclusion, while BwIP-mbPLS shows significant potential in improving predictive accuracy and interpretability in genotype-phenotype mapping, its computational efficiency on larger datasets remains a challenge. Future work should explore methods to optimize its performance and scalability for practical applications involving larger, more complex datasets.
